# A qualitative study on the experiences of southern European immigrant parents navigating the Norwegian healthcare system

**DOI:** 10.1186/s12939-021-01384-8

**Published:** 2021-01-21

**Authors:** Raquel Herrero-Arias, Esperanza Diaz

**Affiliations:** 1 Department of Health Promotion and Development, University of Bergen, Alrek Helseklynge, 7807, Årstadveien, 17, 5020 Bergen, Norway; 2 Child Welfare, Equality and Social Inclusion Research Group, University of Bergen, Bergen, Norway; 3 Department of Global Public Health and Primary Care, University of Bergen, Alrek Helseklynge, 7804, Årstadveien, 17, Bergen, 5020 Norway; 4 Unit for Migration and Health, Norwegian Institute of Public Health, Oslo, Norway

**Keywords:** Immigrants, Norway, Qualitative research, Patient navigation, Primary care

## Abstract

**Background:**

Patients’ experiences with health providers and their diagnostic and treatment expectations are shaped by cultural health beliefs and previous experiences with healthcare services in home country. This study explores how Southern European immigrant parents navigate the Norwegian healthcare system, through its focus on how this group manage their expectations on diagnosis and treatment practices when these are unmet.

**Methods:**

The study had a qualitative research design. Fourteen in-depth interviews and two focus group discussions with 20 Southern European immigrant parents were conducted in 2017 in three Norwegian municipalities. With the help of NVivo software, data were transcribed verbatim and coded. Following a thematic analysis approach to identify patterns in immigrants’ experiences with the Norwegian healthcare services, the codes were organized into two themes.

**Results:**

The first theme includes immigrants’ expectations on diagnostic tests and medical treatment. Southern European immigrants expected more diagnostic tests and pharmacological treatment than what was deemed necessary by Norwegian health providers. Experiences with unmet expectations influenced how immigrants addressed their and their children’s healthcare needs. The second theme comprises immigrants’ experiences of seeking healthcare in Norway (attending medical consultations in the private sector, seeking immigrant healthcare providers, and navigating the healthcare through their Norwegian social networks). This category includes also the alternative solutions immigrants undertook when they were dissatisfied with the diagnosis and treatment practices they were offered in Norway (self-medication and seeking healthcare in home countries).

**Conclusions:**

Cultural health beliefs and previous experiences with healthcare services from home country shaped immigrants’ expectations on diagnosis and treatment practices. This had great implications for their navigation through the healthcare system and interactions with health providers in the host country. The study suggests that successful inclusion of immigrants into the Norwegian healthcare system requires an acknowledgement of the cultural factors that influence access and use of healthcare services. Exploring immigrants’ perspectives and experiences offers important information to understand the challenges of cross-cultural healthcare and to improve communication and equitable access.

## Background

Migration often involves a process of adaptation to function successfully in a new environment [1]. Seeking adequate healthcare for themselves and their children, immigrants learn to navigate the host country’s healthcare system and manage culturally informed expectations regarding diagnosis and treatment practices [2, 3]. Health literacy, or the knowledge, skills and social resources needed “to access, understand, appraise and use information and services to make decisions about health” [4], is a crucial determinant of immigrants’ health and successful navigation through a new healthcare system. In the postmigration context, health literacy includes knowledge of cultural beliefs, the healthcare system and health information channels [5]. Moreover, colearning and social support are practices that promote health information and knowledge, contributing to improving health literacy [6]. Likewise, research has stressed the important role that social networks play in shaping people’s decisions about health [7, 8].

Other factors that influence immigrants’ navigation include their experiences with healthcare services prior to migrating that may act as sociocultural referents when they assess medical practices in the host country [9]. Culture and socioeconomic status also shape immigrants’ strategies to navigate healthcare across countries. In Scandinavia, communication problems and low socioeconomic status are key barriers that immigrants face when accessing healthcare [10–12]; communication is challenged not only by the language barrier but also by divergent cultural understandings of disease, treatment, and the role of the doctor [11, 13–18]. For instance, for Thai women living in Norway and Sweden, culture is a major factor contributing to their unwillingness to access the healthcare system—including, when necessary, by asserting their right to interpretation services [19, 20]. Seeking to overcome their health problems, these women engage in self-treatment, seek care from social networks, and travel to their country of origin for medical consultation; the latter strategy has also been observed among Poles, the largest immigrant group in Norway [12].

Comparative research on immigrant and native populations has found higher expectations for antibiotic prescriptions among Turkish immigrants in Germany [21] and a higher use of antibiotics among non-Western immigrants in Spain and the Netherlands [22, 23]. Research on immigrants’ use of healthcare services has been inconsistent, and while some studies found a higher use of primary healthcare and emergency services among immigrants [24], others indicated the lower use of general practice medical care in the immigrant population [25]. Despite variations among immigrant groups, studies on differences in the use of emergency primary healthcare between natives and immigrants in Norway and Denmark have shown a lower use of services and prescribed medication among the latter [10, 11, 26–28]. A register-based comparative study on primary healthcare service utilization among older immigrants and natives in Norway found that immigrants’ utilization of these services was positively associated with their length of stay in the host country [29]. The study also found higher rates of utilization of primary healthcare services among refugees and lower rates among labor immigrants compared to those among groups who migrated for family reunification.

In Norway, there has been a growing recognition of the risks associated with the unnecessary use of health services and resources [30]. In addition, the country has experienced greater cultural pluralism due to increased immigration and a change in the patterns of migration [31]. Among these patterns is a rise in South-to-North intra-European migration since the 2008-financial crisis.^1^ In this context, it is important to explore immigrants’ expectations regarding diagnosis and treatment, as well as their management of these expectations in their encounters with health providers in Norway. However, research has mainly addressed the experiences of non-Western immigrant groups in accessing Norwegian healthcare. Particularly, the Southern European immigrant community in Norway has remained underresearched compared to more established groups [33].

Aiming to fill this gap, this article explores Southern European immigrants’ expectations of treatment and diagnosis and their management of the divergence between their expectations and the actual medical practices they encounter in Norway, both as patients and as parents of young patients. The article draws on data from a doctoral project that explored how Southern European parents experience parenting and interact with welfare institutions in Norway [34, 35]. Although the informants were not asked directly about healthcare services, their experiences with such services emerged as a key issue in their lives in Norway. We extracted and analyzed the data related to such experiences for the purpose of this article. Understanding Southern European immigrants’ pathways to health will contribute to the improvement of cross-cultural communication and equitable access to healthcare. This understanding is especially relevant in a context in which policymakers and health providers may assume that the immigrant community is unlikely to experience tensions between their expectations and the healthcare they are provided in Norway because they assume that these immigrants are familiar with orthodox healthcare systems.

### Norwegian and southern European healthcare

In Norway, citizens, residents who have lived in the country for longer than six months, and registered asylum seekers are entitled to public primary and secondary healthcare services. Primary care includes care provided by general practitioners (GPs), emergency services, maternal care, child controls, nursing homes, and home care, while secondary care includes care provided by specialists and hospitals [2]. Patients pay a subsidized fee for GP consultations and specialized outpatient care consultations and chronic medication, which caps out when they reach a maximum set by the government (2460 Norwegian crowns). Once this threshold has been met, the system covers all required care for the rest of the calendar year. Residents have the right to choose their GPs and to have an interpreter in their consultations [2, 12].

Despite these policies, there are systemic barriers to communication between immigrants and healthcare providers [36]. E-health and written materials are commonly used to provide health information in Norway, which rely on individuals having language and literacy skills [5]. There are some community-based initiatives providing adapted health information, but these have mainly addressed asylum-seekers and refugees. For labor immigrants coming from European countries, a letter from the Norwegian Health Economics Administration (HELFO) about the GP scheme is the main health information source they access after their arrival.

Portugal, Italy, Spain, and Greece reached similar stages in the development of their healthcare systems in the 1970s and 1980s [37]. This development included the decentralization of services, increased coverage of primary care services, and partial coverage of prescriptions. Southern European healthcare systems are characterized by the important role played by nonprofessional resources, such as families [38]. These healthcare systems experienced detrimental effects of the 2008-economic crisis [39]. Consequently, the Southern European population lost some of its financial capacity and the right to free medication, and privatizations increased [37].

Rates of overuse of medication are higher in Southern Europe (10–30%) than in Northern Europe (5–10%), and the same trend applies to antibiotic resistance [40, 41]. In Spain, a study [42] found that the rate of self-medication was 18.1, and 17.7% of Spaniards used antibiotics without a prescription.

## Theoretical framework

Ungar explored how youth who faced significant levels of adversity navigated mandated services to achieve successful psychological outcomes and how these services could support or hinder youth resilience [43, 44]. He determined that youth navigated health resources and negotiated with service providers in a process in which they identified and accessed the services, structures and relationships required to support their health. Social navigation has also been considered a theoretical concept that illuminates the intersection between the movement of social environments and the movement of individuals [45].

Inspired by the concept of social navigation and Ungar’s socioecological approach, we view immigrants as navigators and negotiators who mobilize the resources they have on hand across countries and negotiate with health providers in their attempts to treat and/or diagnose their and their children’s health problems. The perspective of navigating emphasizes that immigrants assess the available resources and plan different routes into healthcare. We understand negotiation as the process through which immigrants discuss with health providers some aspects of their and their families’ health problems with the aim of accessing the resources and medical practices that they consider effective in overcoming these problems [43]. Changes in employment conditions, insurance coverage, or social networks in both host and origin countries are examples of the moving environment through which immigrants navigate [45].

## Methods

### Study participants

Participants were recruited in different ways: through advertising on Facebook groups used by Southern European immigrants in Norway, the use of RHA’s personal network, the attendance of gatherings organized by immigrant communities in Norway, and snowball sampling.

Because we approached parents raising their children in Norway, the participants were middle-aged adults. The participants included 5 men and 15 women who each had one to three children aged between 9 months and 17 years. The overrepresentation of women in our sample can be explained in terms of the gendered expectations about childrearing [46] and the difficulties recruiting fathers for research on parenting [47]. The participants were originally from Italy (2), Greece (2), Portugal (1), and Spain (15) and had lived in Norway for between three and 14 years (Table 1). None of them had any relationship with the researchers prior to the commencement of the study.

**Table 1:** Sociodemographic characteristics of the informants (*N* = 20)

		Total (n = 20)
**Gender**	Women	15
	Men	5
**Age distribution**	30–35	2
	35–40	7
	40–45	9
	45–50	2
**Country of origin**	Spain	15
	Italy	2
	Greece	2
	Portugal	1
**Civil status**	Single	1
	Registered partnership	4
	Married	15
**Education**	Primary	2
	Secondary	4
	University	14
**Employment status**	Employed	19
	Unemployed	1
**Number of children**	1 Child	8
	2 Children	11
	3 Children	1
**Years lived in Norway**	1–5	7
	5–10	11
	10–15	2

### Data collection and analysis

RHA collected the data in 2017 in three Norwegian municipalities consisting of both rural and urban areas with high concentrations of immigrants. An overview of the data collection methods is shown in Table 2.

**Table 2:** Data collection: number of FGDs, interviews, and participants by country, gender and years lived in Norway

Method of data collection	Country of origin of participants				Gender		Years lived in Norway		Language used	Total participants
	Italy	Greece	Portugal	Spain	Women	Men	< 5	> 5		
**FGDs**										
FGD 1	2	2	0	2	6	0	0	6	English	6
FGD 2	0	0	0	4	4	0	4	0	Spanish	4
**Interviews**										
Individual Interviews	2	2	1	7	9	3	3	9	English (4) Spanish (8)	12^a^
Couple Interviews	0	0	0	2	2	2	2	2	Spanish	4

^a^Among these participants, six (2 Italians; 2 Spanish; 2 Greeks) had previously participated in the FGDs and were interviewed because they raised relevant issues related to their unique situations as single mothers or as women with a Norwegian partner

First, two focus group discussions (FGDs) were conducted at a university setting in Norway. Because including participants of both genders in an FGD can negatively impact group dynamics and because some topics of the discussion, such as parenting, may have been experienced differently by gender, we only included women in the FGDs [48]. We conducted two separate FGDs based on the criterion of length of stay in Norway to ensure the participation of informants who had lived in the country for less time and who might have felt that they had fewer experiences to contribute. RHA moderated the FGDs with the help of a Spanish PhD candidate. Each FGD lasted 120 min and was audio recorded. The participants were asked about their mothering experiences and encounters with Norwegian welfare institutions, including healthcare services.

Second, in-depth interviews were conducted to further explore the topics raised in the FGDs and to verify our interpretations [49]. The interviews lasted 75 to 120 min and were audio-recorded. The interviewees chose the interview location, such as their homes or workplaces. Following a narrative approach that promoted rich descriptions of the context in which experiences were embedded [50], the interviewees were asked about their lives before and after migrating; family backgrounds; transitions; and experiences of parenting and encounters with Norwegian welfare institutions.

RHA transcribed the data verbatim and coded the information using NVivo software. Thematic analysis was conducted guided by Braun and Clarke’s approach [51], with a focus on how the participants talked about their experiences with healthcare services. Based on an iterative approach, the codes and a first organization of themes emerged from the data and were later analyzed in light of the existing scholarship. First, RHA became familiar with the data by rereading the transcripts. Second, she inductively generated codes that summarized the content of each meaning unit and described concepts underpinning the data. Third, these codes were classified under themes according to their similarity of meaning, and the themes were later reviewed and redefined in light of the existing literature. ED contributed important intellectual insights to the review of the final coding tree and the reporting of results.

### Methodological considerations

Qualitative methods allowed the exploration of a field of study that had been relatively underresearched and the inclusion of immigrant groups that are usually not addressed in the Scandinavian literature. Methodological triangulation enhanced the data credibility. Moreover, triangulation through the interpretation of the data by researchers with backgrounds in anthropology and medicine and the inclusion of quotations from the informants strengthened the quality of the study.

### Reflexivity

The positionality of the researchers may have influenced the research [52]. As a Spanish researcher in Norway, RHA had an “insider” status that facilitated trust building, especially with the Spanish participants. For the rest of the participants, RHA may have been considered an “insider by proxy” [53], as she was an immigrant researcher from another country. Because she came from a country other than Greece, Portugal or Italy, these non-Spanish participants assumed she was not very familiar with the realities of their countries and explicitly explained aspects related to their societies and healthcare systems. In both cases, RHA’s insider status facilitated the data collection because all participants felt comfortable sharing their critical opinions about Norwegian healthcare based on the assumption that she would not feel offended because she is not part of the majority (Norwegian) society. The participants also presumed that RHA had an understanding of their migration experience and knowledge of stereotypical views on Southern European immigrants.

However, the insider status may have led to bias. For instance, the participants often made assumptions about RHA’s insider knowledge of the Norwegian and Spanish healthcare systems. When this happened, RHA asked for further elaboration on the topic. Finally, the fact that ED, who has extensive expertise in medical research and practice in Norway, participated in the data analysis mitigated the potential limitation created by RHA’s lack of knowledge about healthcare provision in Norway.

## Results

Two themes emerged from the data: immigrants’ expectations concerning diagnosis and treatment and pathways to healthcare (Table 3).

**Table 3:** Presentation of themes and sub-themes

Themes	Sub-themes
Expectations concerning diagnosis and treatment	• Expectations concerning diagnosis • Expectations concerning treatment
Pathways to healthcare	• Seeking healthcare in the private sector • Seeking immigrant health providers in Norway • Navigating healthcare through Norwegian social networks • Negotiating with Norwegian health providers in Norway • Alternative strategies for treatment and diagnosis

### Expectations concerning diagnosis and treatment

#### Expectations concerning diagnosis

The participants expected healthcare providers to perform tests to diagnose health complications at an early stage and to conduct screenings in order prevent sickness. They also expected health providers to possess the type of vast medical knowledge that would lead to an accurate diagnosis. When these expectations were unmet, they felt frustrated and dissatisfied with the healthcare services.

*My daughter had chicken pox. We thought so; it wasn’t like the doctor thoroughly checked her to see what she was suffering from. (Spanish mother, FGD 2).*

*[…] Doctors lack basic knowledge of pediatrics. They don’t do any [diagnostic] tests either. How can you trust them? (Portuguese father, individual interview).*

Although the participants prioritized the use of diagnostic tests as a measure to prevent health concerns, the participants who had lived in Norway for a longer period of time reflected on the risks of overdiagnosis, a practice they linked with their countries of origin.

*There are both bad and good things about that (fewer diagnostic tests), because in Greece, it’s like, “I have [something in] my head, I run to the doctor and.…” (Greek mother, FGD 1)*

*Have an X-ray. (Italian mother, FGD 1)*

*“… you get crazy.’ Whereas here, because you know it’s not that easy to go and check your finger, then ‘OK, maybe it’s not something serious.” (Greek mother, FGD 1).*

To make sense of the less interventionist healthcare approach encountered in Norway regarding diagnostic practices, the participants pointed out the authorities’ intention to save economic resources. They also reported that the use of diagnostic tests would prevent possible problems:

*When I got pregnant, well, in Spain, you usually take blood tests to see your sugar levels. Here, I called [and said], “I want an appointment because I think I’m pregnant. I want to confirm that and maybe take some tests.” “OK, you are younger than 37, so you don’t have to come until week 14.” “But aren’t you going to check if I’m fine?” “No, because miscarriage can happen during the first trimester.” (Spanish mother, FGD 1)*

*But what they say is true. (Spanish mother, FGD 1)*

*But it’s stressful when you come from another country. (Italian mother, FGD 1)*

*It’s just to save money to the system, which is great […]. (Spanish mother, FGD 1)*

*But a lot of things can be prevented. (Italian mother, FGD 1)*

*That’s true as well. (Spanish mother, FGD 1)*

Another factor that reinforced distrust toward the diagnostic practices encountered in Norway was the medical devices used, which the participants considered to be low-tech. This perception was especially strongly voiced in the accounts of the participants who had given birth in Norway and shared their experiences with prenatal screening and the use of noninvasive devices such as the Pinard stethoscope:

*[During pregnancy], the check-ups were very funny, “I check the belly, the heart, with a piece of wood,” very rudimentary. (Greek mother, individual interview).*

Few participants reported that health providers had explained to them why fewer diagnostic tests were conducted in Norway than in their countries of origin. The participants usually appreciated such explanations as a practice that helped them understand the diagnostic practices in Norway.

*[…] I just told the doctor I was worried because in Greece, I‘d take […] something like an ultrasound every month, or every two months. She [the doctor] explained to me that ultrasounds are much safer than X-rays, but we don’t really know its effects in the longer term. […] This conversation calmed me down. I understood why they do things the way they do. It wasn’t random; they have their reasons. (Greek mother, individual interview).*

#### Expectations concerning treatment

When evaluating their encounters with health providers, the participants compared their experiences with those in Southern Europe. Most of them found that GPs in Norway prescribed little more than mild painkillers instead of other pharmacological treatments that the participants deemed more effective, such as antibiotics. As shown in the subsequent quotes, the participants did not associate practices such as resting, drinking water, or taking painkillers, as suggested by GPs, with effective treatment. These perceptions were reflected in the data, especially in the accounts of the participants who had lived in Norway for less than 10 years.

*There isn’t a need to go there [to a consultation]. You go and they say, ‘Paracetamol for children.’ (Spanish mother, FGD 2)*

*It’s terrible. GPs do nothing […] they just speak to you. (Spanish mother, FGD 2)*

*They solve everything with water. (Spanish mother, FGD 2)*

The lower frequency of the prescription of antibiotics was understood as part of a more ‘natural’ approach that guides Norwegian healthcare providers.

*We are very interventionist: “I give you a medicine, I cure you, I monitor you.” Here, that doesn’t exist. In Sweden, Germany, and Denmark, it’s the same because the concept of health is different. Their approach is more natural, and this has a positive side because they don’t intervene when it isn’t necessary, but it’s scary for us, because it’s a new approach. (Spanish mother, FGD 2).*

This clash in the different countries’ prescription practices was more evident to the participants in consultations for their children.

*Don’t ever think of taking a baby (to the doctor) and asking for medication, because they’ll look at you as if you came from Mars. (Spanish mother, FGD 2).*

Although the participants acknowledged the advantages of the Norwegian approach (avoiding an increase in antibiotic resistance and reducing the number of unnecessary treatments that may cause harm), they also considered this approach a potential threat to their health and that of their children. This was especially the case when children had fever or infections, as the participants believed antibiotics were the only effective treatment to overcome these problems.

*They (doctors) think things are cured naturally. They don’t intervene a lot […] which is good to some extent: children’s immune system, not taking too many antibiotics… but if the child has patches of pus, you must intervene, and they don’t! (Spanish mother, Individual interview).*

Statements such as “the concept of health is different” or “doctors think things are cured naturally” show that the participants understood the divergence in treatment practices from a cross-cultural perspective; that is, they understood treatment practices as being shaped by contrasting cultural health beliefs. The participants’ expectations about pharmacological treatment also mirrored their beliefs about health providers’ competence and roles. The participants did not approve of GPs who could not tell them immediately which dose of medication their children should take without consulting the National Treatment Guidelines for Health Personnel or the Internet. This clash between their expectations of a competent GP and adequate pharmacological treatment versus the practices they encountered led to feelings of dissatisfaction, frustration, and abandonment.

*He (the GP) prescribed me ibuprofen. I asked him about the dose, […] he looked at the directions for use on his phone […] (Spanish mother, FGD 2)*

*[…]*

*A doctor always knows! In Spain, we trust doctors because they act with determination and wisdom (Spanish mother, FGD 2)*

*[…] […] You get a feeling of abandonment, as if you aren’t being cared for. (Spanish mother, FGD 2).*

The lower frequency of the prescription of antibiotics was also described as a sign of health providers’ lack of competence and knowledge, especially regarding an accurate diagnosis, effective treatment, and pediatrics.

*Doctors are afraid (to intervene) because they don’t have the knowledge. […]‘A child! I’m very afraid to prescribe antibiotics.’ (Portuguese father, individual interview).*

### Pathways to healthcare

Having experienced a clash between their expectations and medical practices, the participants navigated the Norwegian healthcare system and negotiated with health providers with the aim of receiving the desired care for themselves and their children. When their efforts were not successful in having their expectations and needs met, the participants sought alternative solutions. The following subthemes describe the strategies that the participants employed in that process.

#### Seeking healthcare in the private sector

For those informants who could afford them, Norwegian private healthcare services were an alternative to the diagnosis and treatment practices offered by the public services. This strategy was especially identified in immigrant mothers’ accounts of their experiences with maternal healthcare services in Norway.

*When I was pregnant, I wasn’t worried, because I had an ultrasound in the private clinic, and it was OK. (Spanish mother, individual interview).*

The participants made use of the private sector to have tests that were not offered by Norwegian public healthcare services or when they wanted a second opinion. An example is provided in the next quote, in which a father shared his experience of seeking healthcare for his child who was sick with laryngitis.

*We (my wife and I) went to a private doctor. We wasted our time because […] the treatment offered in the public hospital was so preventive. It took a month until my son was prescribed with a corticoid! The public hospital has a therapeutic approach that is completely conservative, whereas hers (the private doctor’s) was more interventionist against the illness, more aggressive but suitable and effective. (Portuguese father, individual interview).*

#### Seeking immigrant health providers in Norway

The participants expected that immigrant healthcare providers would provide better healthcare because they would have similar understandings of the ‘proper’ treatment, that is, beliefs about the appropriate medical practices to overcome a health problem.

*When I knew I could change my GP, I looked for an Eastern European or Latino name because I guessed immigrant GPs would understand us (immigrants) better. They wouldn’t look surprised if I disagreed with paracetamol as the best treatment (Spanish mother, individual interview).*

Having a rich social network in Norway that would allow access to immigrant healthcare providers was seen as a resource that would facilitate navigation through the Norwegian healthcare system.

*I was lucky because a friend of a friend is a gynecologist who is also an immigrant. When I had problems and was given a very late appointment, I got in contact with him. […] “I think that date is too late for a check-up. I’m going to see what I can do. I’ll speak to somebody.” […] You need to know people. (Spanish mother, individual interview).*

#### Navigating healthcare through Norwegian social networks

Another strategy the participants employed was to approach Norwegian relatives or friends who had more experience and knowledge of healthcare services and to ask them for favors. This was seen as a viable strategy because of immigrants’ assumptions that, unlike them, locals did not face cultural barriers when communicating with health providers. Furthermore, the participants’ lack of knowledge about their rights and the organization of healthcare services was another potential barrier to healthcare. However, their Norwegian acquaintances had more knowledge and experience, as they had lived in the country longer. Having a network of Norwegian contacts could also translate into having a network of known health providers, which could facilitate seeking healthcare.

*My wife’s auntie knows some doctors, and this really helps and calms you down, because […] she knows how to ask for things with the Norwegian touch, because in many cases, the Spanish touch collides with the Norwegian one. (Spanish father, couple interview).*

*Doctors will listen to her because they speak the same language, and I’m not talking just about Norwegian, but […] she knows they don’t intervene a lot; then, if she asks for that test to identify Down syndrome during pregnancy, the doctor won’t think that she is a fussy immigrant pregnant woman. (Spanish mother, couple interview).*

This Spanish couple shared their opinions of Norwegian healthcare services that do not offer frequent prenatal visits to monitor a pregnancy. In their attempts to obtain the expected diagnostic tests, they approached a Norwegian relative who “speaks the same language” as providers. The couple made it explicit that speaking the “same language” also refers to having knowledge about culturally appropriate communication in the context of healthcare consultations.

#### Negotiating with health providers in Norway

The participants shared their experiences of negotiating medical practices with health providers in Norway. An example of this is the experience of a Spanish mother who described a GP consultation with her daughter and how the GP negotiated prescription practices with her.

*I said [to the GP], “Look, she has ear pain, […] maybe she needs antibiotics.” “Well, she might, she might not. I’m going to leave it prescribed in the pharmacy; if you see that the girl gets worse in five to seven days, pick up the antibiotic, but if she gets better, the antibiotic prescription will expire.” (Spanish mother, FGD 2).*

The GP’s described action can be seen as an attempt to “meet in the middle”, to offer a treatment that would leave both parties satisfied. However, as we show next in the subtheme ‘alternative strategies for treatment and diagnosis,’ this participant did not see this approach as an effective solution to her daughter’s needs but as a waste of time that confirmed the pointlessness of consulting Norwegian health providers and her need to look for an alternative solution.

Despite this experience, most participants appreciated the efforts that health providers made to involve them in decision-making, which positively influenced their opinions about Norwegian healthcare services.

*You have a lot of decision-making capacity. It’s really good […] In Spain, it’s a bit more like you get there [to the hospital] and are treated like a patient, as if you are sick: “Come here, sit down, don’t.” Here you are listened to more. (Spanish father, individual interview).*

#### Alternative strategies for treatment and diagnosis

The participants navigated conflicting healthcare practices and expectations by employing whatever resources they had on hand to receive the care they needed or expected. In many cases, they refused to use healthcare services in Norway and instead brought medications from Southern Europe for unsupervised treatments for themselves and their children.

*I came out of the consultation and said to my daughter, “See? We shouldn’t have come. I have medication at home. I’m going to cure you.” (Spanish mother, FGD 2).*

*If I say, “My daughter needs treatment,” the GP will look at me as if I were overreacting because I’m from the South, “where people are so dramatic.” I have medication at home. I can help my daughter. (Spanish mother, FGD 2).*

*I bring everything [medication] from Spain. I worked in a pharmacy; I know them [pharmacists], and they give me things. (Spanish mother, individual interview).*

Traveling to their countries of origin to access care for nonurgent health concerns and diagnostic tests was also a common practice among participants.

*If it isn’t that serious, you wait until holidays to consult about whatever you are concerned about with your doctor [in Spain]. (Spanish mother, FGD 2).*

## Discussion

The Southern European immigrants in this study expected more diagnostic tests and pharmacological treatment than Norwegian health providers considered necessary. This clash between expectations and medical practices was reflected across the data, regardless of the participants’ age, gender, and level of education. Inspired by the work of Ungar and Vigh [43–45], we argue that immigrants navigated the available resources and negotiated with health providers’ treatment and diagnostic practices for themselves and their children, which resulted in different pathways to healthcare.

The participants described their experiences with Norwegian healthcare services as reflecting a cultural clash. They referred to divergent culturally shaped beliefs of health and sickness that underpinned healthcare services and practices between their host and origin countries. Understanding that culture influences medical practices and health beliefs, the participants assumed that immigrant health providers would give better healthcare because of their shared notions of appropriate treatment and diagnosis. As shown by a study conducted in the Netherlands [54], contrasting explanatory models of health and sickness between patients and health providers may challenge intercultural communication in general practice. In this regard, the participants in our study expected health providers to intervene to restore their health and believed in the power of medicine to help these professionals in this task. A study on Poles in Norway [12] found that such expectations resulted in dissatisfaction with health providers when these professionals referenced literature or could not provide an accurate diagnosis at an early stage. In line with a study on immigrant parents of children with disabilities in Norway [13] and based on our data, we argue that immigrants’ expectations might be influenced by their experiences with health providers in their home countries who exercise professional authority through medical expertise. The participants talked about health providers in Southern Europe as being respected for having vast knowledge that legitimizes their medical interventions.

Cultural and language differences, socioeconomic factors, social support and the composition of social networks influence immigrants’ health literacy skills, including their ability to access and assess information to make health decisions and, therefore, their possibilities to navigate the healthcare system. Tensions between culturally shaped beliefs and communication styles may challenge the establishment of a relationship based on trust and empathy with health providers [6]. The participants perceived that health providers underestimated their concerns about their and their children’s health because they expressed those concerns in a way that was influenced by their culture, which is consistent with previous literature [55, 56]. They also stated that Norwegian health providers categorized them as ‘overdramatic’ and held stereotypes about immigrants that made it difficult for them to express themselves freely [13, 57].

Consistent with a previous analysis [58], the participants assumed that they had to rely mostly on the relations within their immigrant communities that they trusted (bonding capital) [7], which may have further translated into limited access to trustworthy information about local healthcare and misconceptions about the services offered. To improve individuals’ health literacy, creating spaces where health providers and patients can share information and critically reflect on their own knowledge is important [6]. Such spaces would promote bridging social capital [7], a positive resource that can support immigrants’ navigation. The importance of bridging social capital was evidenced by the participants’ descriptions of communicating with health providers through Norwegian acquaintances who could express themselves in a culturally competent way. Bonding social capital emerged as a resource to contact and negotiate with immigrant health providers. Both strategies highlight the important role of social support and networks in immigrants’ navigation through healthcare systems [8, 59].

The Southern European immigrants were dissatisfied with Norwegian health providers’ attitudes and practices, especially when these professionals were reluctant to prescribe antibiotics and additional diagnostic tests and believed in the body’s power to heal itself [12, 60]. This finding shows that the participants equated pharmacological treatment with receiving ‘proper’ treatment and being taken seriously by doctors, and the lack of pharmacological treatment created a barrier to their use of healthcare [12, 61]. Furthermore, their experiences with healthcare services in their countries of origin that suffered the detrimental effects of the 2008 recession influenced their interpretations of Norwegian healthcare providers’ reluctance to prescribe antibiotics. Rather than seeing the reticence to prescribe antibiotics as an approach that avoids risks associated with unnecessary treatment, the immigrants interpreted it as motivated by economic shortages and attempts to save money.

Difficulties in obtaining antibiotic prescriptions and diagnostic tests prompted the participants’ decisions to medicate themselves and their children and not to seek healthcare in the host country. This finding contrasts with a study that found lower antibiotic consumption in immigrant children than in Norwegian children, which included only prescriptions bought in Norway [62]. A potential explanation for these contrasting findings may be increased self-medication. Our participants described practices of bringing medicines from Southern Europe for unsupervised treatment for themselves or their children and seeking healthcare in their countries of origin. Such practices have also been noted among other immigrant groups [12, 19].

In September 2018, the Norwegian Medical Association launched a ‘Choosing Wisely’ campaign, which is in line with global initiatives to prevent risks from overdiagnosis and overtreatment, such as the unnecessary use of resources, antibiotic resistance, and harm to patients who do not need such intervention [30, 63]. Based on our data, we argue that Norwegian health providers seem to have followed updated guidelines that promote public health and the good use of resources when they met the participants in our study. However, in our study, health providers, by implementing guidelines without considering the cultural context of their interactions with immigrant patients, may have reinforced self-medication and the avoidance of consultations, even when the intention was the opposite. Measures designed to facilitate negotiation with patients and, consequently, patient satisfaction, such as *vent-og-se-resept* (delayed prescription), were counterproductive when cultural differences were neither explicitly acknowledged nor negotiated.

On the other hand, the participants highly valued being regarded as competent informants who could identify signs of disease and who could give an opinion about suitable treatment. Welcoming the opportunity to participate in decision-making is common among immigrants coming from countries where health providers have more authority [13]. Likewise, the informants appreciated that health providers explained the reasons for the diagnostic and treatment practices offered in Norway instead of just criticizing other approaches. When doctors provided such explanations, the participants reflected on the benefits of the medical practices encountered in the host country. Positive interpretations and appreciations of the treatment received in Norway were more common in the accounts of those who had lived longer in the country, which also shows immigrants’ adaptation process [23].

### Limitations

Rather than aiming for generalization, qualitative research provides a rich and contextualized understanding of human experience. We have contextualized the findings to help readers assess whether the results might be relevant and transferable to other groups, i.e., to immigrant groups with similar backgrounds. A potential limitation of the study may be that while we presented the participants’ reflections on their encounters with health providers, we did not examine the experiences of health providers. Further research would benefit from interviews with both immigrants and health providers about the same experiences, i.e., treatment and diagnosis, to generate a better understanding of cross-cultural healthcare. Likewise, a participatory research approach engaging immigrants and health providers could promote colearning, understanding and recognition of diverse knowledge and opinions [6].

Although the sample was diverse in terms of age, gender, and occupation, a majority of the participants were highly educated, and only five were men. Level of education and gender may be factors that shape patients’ experiences, expectations, and perceptions. We did not identify such differences, yet a larger study would provide further knowledge about the influence of such variables related to patients’ perspectives.

## Implications and conclusions

The Southern European immigrants in this study expected more diagnostic tests and pharmacological treatment than their Norwegian GPs considered necessary. Influenced by their cultural beliefs on health, their expectations of medical intervention, their expectations of the GP’s role and competence, and their available resources, immigrants traveled several pathways to secure healthcare for themselves and their children.

Our study has important implications for research and practice in light of the worldwide focus on overdiagnosis and overtreatment. Health providers are in a position to bridge the gap between scientific knowledge and patients’ expectations by raising awareness about the risks that medical overintervention poses to individuals and healthcare services. However, as our study suggests, such efforts should be carried out in a culturally appropriate manner. Our study calls for further exploration of how immigrant patients navigate healthcare in Norway in the context of the ‘Choosing Wisely’ campaign implemented to reduce overdiagnosis and overtreatment. This knowledge would promote an awareness of cultural differences and expectations regarding diagnosis and treatment among health providers, which is crucial to improve negotiation of medical activity and navigation of the healthcare system.

## Data Availability

The dataset generated and analyzed during this research study is not publicly available because the participants did not consent to the data being made available to other researchers. However, additional qualitative data from our dataset are being published elsewhere.

## Footnotes

1 In 2018, Spaniards comprised the largest Southern European immigrant community in Norway, with 6211 members, followed by the Italian (4315), Portuguese (3218), and Greek (2828) [32].
